# Supplementary material for: Chromothripsis during telomere crisis is independent of NHEJ, and consistent with a replicative origin
Source: Genome Res. 2019 May;29(5):737–49. doi: 10.1101/gr.240705.118 (PMC6499312; doi:10.1101/gr.240705.118)
Supplement: Supplemental Material [file supp_gr.240705.118_Supplemental_file_1.zip › contigs/annotated_contigs/DB115/contig.2.DB115_length_592_mean_cov_7.09459459459.docx]

**DB115_length_592_mean_cov_7.09459459459**

```
GATACCTAGTTGTCCCATCAGCATTTATTGGAAAGAGTGTCATGTCTCCACCGCATTGTCATCTTTGTCATAAATCAGGTGACTGTGTA
 >chr19:30438904-30439301 + E=3e-224
AGTAGATCAGTTTCTTGACTGTTCTGTTGGCTTGTCTGTTCTTGCAGCAAACCTACACTGTCTAAATCATTATGACTTTGTGTTATCTT

GGTACCTGGTACATGGTGGTGTAGTTTTAGTTGTGGTTCCTCAAGATTGCCTTTATTTTCTCCTTTCCAGTCCGTATGCTGTTTCTTTC

TTTTTCTTGCTTTTGATACTTGCCAGTTCAAGTGCTGGTAGTGGGCATCAGGGGTCAACTTTTAAACATTTACCATTAAGTGTGGTATT

TACTGTGAGTAATTTTTGTATTTGCTTTTATCAGATTAAGG|CA|TCTTTCTGTAGCTGGATAACTCTTAGATCTGATTCATCAACCTG
                                            >chr19:30447289-30447482 + E=2e-104
CTGAACAGTTCCTTTTTCAGAGACATAGATACCATCCAAAAATTTCCTGATATCCTTGTTTTTAACTGTTGTGGGTTGCTGAATCAAAG

CTGCTGAATTTGAAACAAGCTCAGTGTCATTTCCTTCAAGGATTAATTCATCTTTCTGGG
```
